# Study the Expression of *marA* Gene in Ciprofloxacin and Tetracycline Resistant Mutants of *Esherichia coli*

**Published:** 2013

**Authors:** Razieh Pourahmad Jaktaji, Rayhaneh Ebadi

**Affiliations:** a *Department of Genetics, Faculty of Science, University of Shahrekord and Biotechnology Center, University of Shahrekord, Shahrekord, Iran. *; b *Genetics at University of Shahrekord. *

**Keywords:** *acrAB* operon, *gyrA* mutants, *marA* gene, *marR* mutation

## Abstract

MarA activates two membrane dependent mechanisms of resistance to different antibiotics, such as ciprofloxacin and tetracycline, including promotion of outflux and inhibition of influx of antibiotics. Thus, MarA causes multiple antibiotic resistance phenotype. The activation of these mechanisms needs overexpression of *marA*. This could happen through mutation in *marR*. Thus, the aim of this study was to measure *marA *expression in ciprofloxacin resistant *E. coli gyrA *mutants and clones with or without *marR *mutation. For this purpose, real time PCR was used to measure relative expression of *marA *in above mutants and clones. Results showed that two clones, C14 and C17 overexpressed *marA*. It is concluded that the level of *marA *expression is important for activation of above mechanisms.

## Introduction

**Figure 1:**
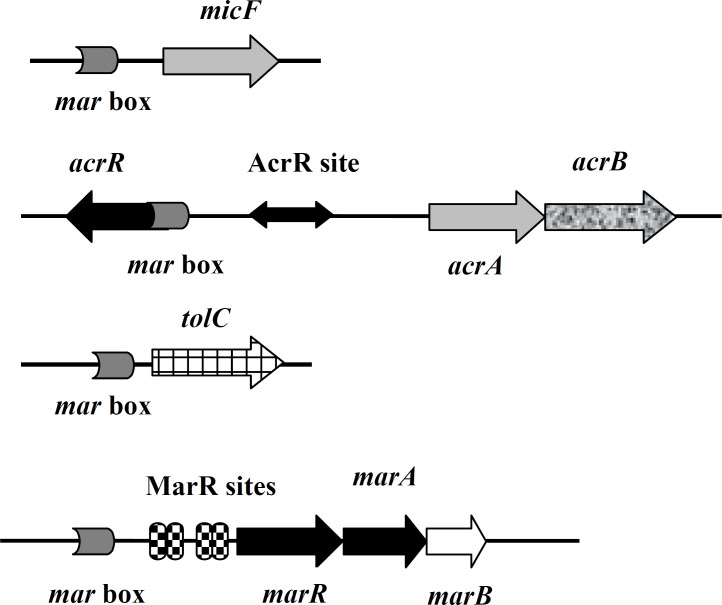
Operons and genes under the positive control of MarA and negative control of AcrR and MarR. Modified and adapted from Dzwokai *et al* (12).

MarA is a member of the AraC family of transcriptional activators found in gram negative bacteria, including *E. coli *(1, 2). It activates its own transcription and that of *mar *regulon genes by binding as a monomer to 20 base pair asymmetric sequences known as marboxes via its N-terminal helix-turn-helix (HTH) motif involved in interaction with DNA (3, 4). These marboxes are located upstream of -35 site of the promoters of target genes (5). One of these target genes is *micF*, which produces an antisense RNA (Figure 1) that downregulates the expression of *ompF *gene by base pairing to its mRNA (6). *ompF *encodes an outer membrane protein called OmpF (7). This is a drug entry site for fluoroquinolones (8). Members of this family of antibiotics, such as ciprofolaxin are the antibiotic of choice for treatment of *E. coli *infections (8, 9). 

The other examples of MarA target genes are *acrAB *operon and *tolC *which altogether encode a membrane three component efflux transporter called AcrAB-TolC (10). This extrudes drugs such as ciprofloxacin and tetracycline out of cells (10). AcrR is the repressor of *acrAB *operon (11). Its encoding gene is located upstream of this operon in the opposite direction (Figure 1). 

Taken together, downregulation of OmpF and upregulation of AcrAB-TolC by overexpression of MarA make the cells resistant to multiple irrelevant antibiotics, including ciprofloxacin, tetracycline and chloramphenicol (3, 13, 14). 

Normally, *marA *expression is suppressed by MarR which is encoded by *marR*. This along with *marA*, is a member of *marRAB *operon. MarR binds as a dimer to two sites (site I and site II) in operator region of this operon (marO) which is downstream of marbox (Figure 1). Site I is located between -35 and -10 region while site II is near translation start site. Site I is important for repression as deletion of it leads to MDR phenotype (15). 

Attachment of different ligands, such as antibiotics to MarR repressor dissociates it from the operator site of *marRAB *operon (16). Then, binding of MarA to mar box activates the expression of *marRAB *operon (4, 5). Overexpression of *marA *could happen via mutations in *marR *(3, 15). In the previous study we described *gyrA *mutants with or without a mutation in *marR *(17). These mutants were ciprofloxacin and slightly tetracycline resistant. Their derivative clones posses intermediate levels of resistance to tetracycline (submitted for publication). To understand whether or not *marA *overexpresses, the level of *marA *expression was measured by real time PCR in these mutants and clones. 

## Experimental

*Antimicrobial agent and chemicals*

Tetracycline hydrochloride (Tc) was obtained from Sigma to induce MDR phenotype. Stock solution was 4 mg/mL.

*Bacterial strain and mutants*

MG1655 was parent strain. *gyrA *mutants with or without a mutation in *marR *gene isolated in previous work (17) are listed in Table 1. 

**Table 1:** Bacterial strain and mutants

**Strain/Mutant/Clone**	**Relevant properties**	**MIC**		**Source/Reference**
		**Cip (µg/ml)**	**Tc (µg/ml)**	
MG1655	Wild type	0.035	3	A gift from Prof. R. G. Lloyd
W25	Wild type; *gyrA* (Ser_83_→Leu) and *marR* (Met_74_→Thr)	0.075	4	(17, 18)
W26	Wild type; *gyrA* (Ser_83_→Leu)	0.075	4	(17, 18)
W49	Wild type; *gyrA* and *marOR* (20 bp duplication in operator)	0.625	4	(17, 18)
C6	W25; selected on tetracycline (5 µg/ml)	1	45	Submitted for publication
C14	W26; selected on tetracycline (5 µg/ml)	1	30	Submitted for publication
C17	W49; selected on tetracycline (5 µg/ml)	1	30	Submitted for publication

Cip and Tc are abbreviations for ciprofloxacin and tertracycline, respectively

As mentioned previously mutants W25, W26 and W49 were isolated from cultivation of wild type strain on LBA containing ciprofloxacin (18). Clones C6, C14 and C17 were derived from cultivation of above mutants on LBA agar in the presence of Tc (submitted for publication). It was explained that resistance to ciprofloxacin can be divided to three levels, including low levels of resistance (MIC: 0.063 to 1 μg/mL), intermediate levels of resistance (MIC: 1 to 32 μg/mL) and high levels of resistance (MIC: >32 μg/mL) (19). Additionally, It was described that resistance to tetracycline can also be divided to three levels, including low levels of resistance (MIC: 1 to 10 μg/mL), intermediate levels of resistance (MIC: 10 to 50 μg/mL) and high levels of resistance (MIC: >50 μg/mL) (20). Based on above definitions mutants have low to intermediate levels of resistance to ciprofloxacin and tetracycline. 

*Media*

LB broth (Merck) and LBA containing 1.5% agar (Merck) were used for cultivation of strain and mutants. 

*Expression analysis of marA*

Real time PCR was used to quantify gene expression of *marA *and *gapA *as housekeeping gene. Overnight cultures on LB broth were grown on LB broth plus 3 μg/mL Tc (except for wild type) at 37ºC with shaking at 150 rpm to mid-logarithmic phase (OD_600_ of 0.6), as described previously (13, 21). Before extraction of total RNA, each culture was stabilized by RNA protect bacterial reagent (Qiagen, Germany) and then pelleted by centrifugation (Sigma, Germany). RNA was extracted immediately after lysozyme-proteinase K digestion of bacteria using an RNeasy Mini Kit (Qiagen, Germany). Contaminating genomic DNA was digested by RNase-free DNase I (Fermentas, Life science research) according to the manufacturer’s instruction (Fermentas, Life science research). RNA purity and concentration was estimated at OD_260_ by spectrophotometer (Ultrospec 1100, Amersham Pharmacia Biothech). Reverse transcription was conducted using the RevertAid Reverse Transcriptase kit (Fermentas, Life science research), random hexamer and Purified total RNA (2 μg). The negative controls without reverse transcriptase were used to confirm the lack of contaminating DNA in the RNA samples. The cDNAs obtained from reverse transcription and negative controls were amplified by PCR reaction to first verify that negative controls do not produce PCR products and second to find the best annealing temperature for real time PCR. Then, diluted cDNA (2 μL of a 1:10), obtained from reverse transcriptase, were used to quantify the level of *marA *and *gapA *with specific primers as mentioned in Table 2 by real time PCR in a Rotor Gene 6000 thermocycler (Corbett Research, Australia) using a SYBR Green kit (Takara, Japan). Serial dilutions of *marA *and *gapA *cDNAs were used as standards in real time PCR reactions. Thermal cycling conditions were described previously (21). Relative gene expression was calculated using the efficiency method pfaffl (ratio of *marA *expression to *gapA *expression) (22). All data on *marA *expression are the average of triplicate analyses. The data were recorded as mean ± SD.

**Table 2:** List of primers

**Reference**	**Length of amplicon (bp)**	**Primer sequence (5′-3′)**	**Gene**
(21)	187	CATAGCATTTTGGACTGGAT	*marA*
		TACTTTCCTTCAGCTTTTGC	
(21)	170	ACTTACGAGCAGATCAAAGC	*gapA*
		AGTTTCACGAAGTTGTCGTT	

*Statistical analysis*

Statistically significant differences in gene expression were determined by Student’s t test (two paired samples, with two tailed distribution), using SPSS version 16 software.

## Results

**Figure 2:**
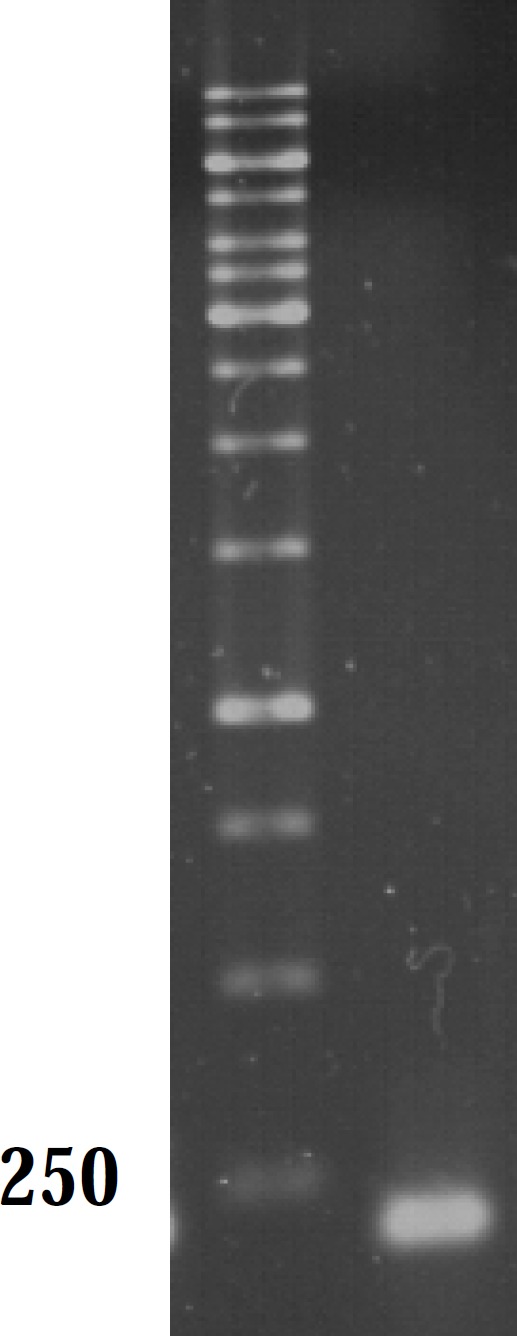
Gel analysis of PCR product. Lane M and A contain 1 kb DNA ladder and *marA* PCR product, respectively

**Figure 4:**
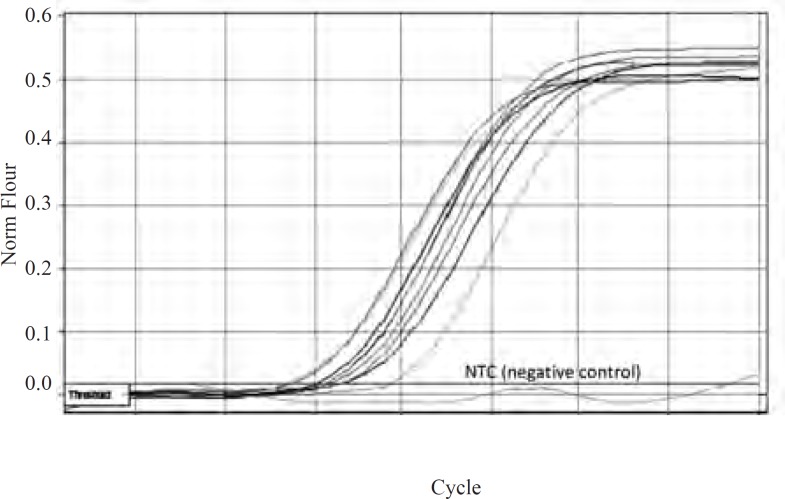
Amplification curves of *marA* in wild type and mutants. The pale blue curve belongs to wild type and other colored curves belong to mutants

As mutations used in this study harbor either or not a mutation in *marR*, it was possible that they increase *mar*A expression. All negative controls without reverse transcriptase did not show bands on gel after PCR amplification and gel electrophoresis. Thus, all RNA samples were purified and without DNA contamination. They were used for real time analysis. The suitable annealing temperature was 52˚C as the result of gel electrophoresis following PCR reaction showed (Figure 2). Data derived from standard curves of *marA *and *gapA *showed that the efficiency of *marA *and *gapA *were 1.92 and 2.1, respectively and coefficient of determination (r^2^) of *marA *and *gapA *were 0.996 and 0.999, respectively. The melting curve of two genes showed just one major peak which indicates the purity of samples. Figure 3 shows the melting curve of *marA*. The melting point of *marA *and *gapA *was 86 degree centigrade. Figure 4 shows the amplification curve of *marA*. Cts (treshhold cycles) of control strain and mutants obtained from this figure and amplification curve of *gapA *used to calculate relative *marA *expression. Table 3 shows the *marA *relative expression in these mutants. The T-test analysis showed no significant difference between wild type and original mutants for expression of *marA *(p < 0.05). The reason for this result may due to low level of resistance to Tc. However, for two clones (C14 and C17) this comparison showed significant difference (p < 0.05).

**ّFigure 3:**
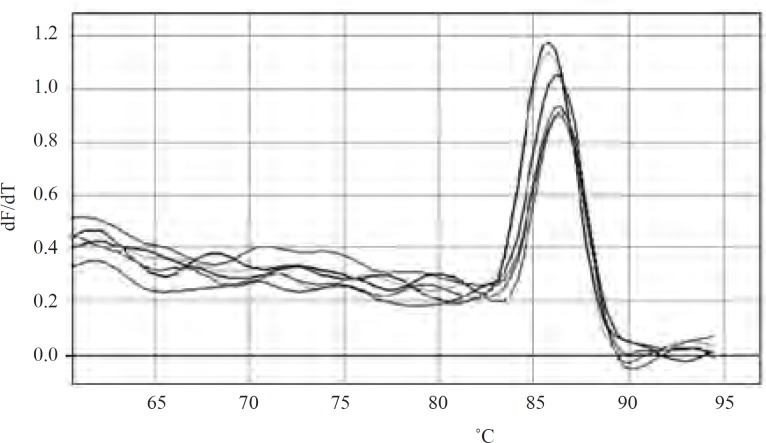
Melting curves of *marA* in wild type and mutants. The yellow color curve belongs to wild type and other colored curves belong to mutants

**Table 3:** Relative expression of *marA* in wild type (MG1655) and mutants as determined by real time PCR

**Strain/mutant/clone**	**Relative expression**
Wild type (MG1655)	1±0
W25	0.9±0.04
W26	0.92±0.02
W49	0.956±0.02
C6	0.97±0.015
C14	2.83±0.05
C17	3.21±0.04

Expression relative to MG1655, mean values from three independent experiments. Figures are the ratio of gene expression between the target gene (*marA*) and the reference gene (*gapA*). An effect on gene expression was considered significant when the corresponding ratios were >2 or <0.6 with a P value of less than 0.05.

## Discussion

MarA regulates two membrane dependent mechanisms of resistance to fluoroquinolones and also other structurally irrelevant antibiotics (14). These mechanisms cause multiple antibiotic resistance phenotype and mainly associated to decreased entrance of antibiotics because of low synthesis of OmpF and extrusion of drugs via over activation of AcrAB-TolC pump (14).

Normally, the expression of MarA encoded by *marA*, is inhibited by MarR repressor (4). However, mutation in its encoding gene, *marR*, alleviates this suppression (15). In the previous study, it was revealed that some *gyrA *mutants with different MIC for ciprofloxacin had either or not a mutation in *marR *(17). These mutants and their derivative clones were more or less resistant to tetracycline as well. To study the contribution of MarA regulator to ciprofloxacin and tetracycline resistance, the level of *marA *expression was measured by real time PCR in these *gyrA *mutants and clones.

It was shown that the binding of ligands, such as Tc to MarR leads to dissociation of MarR from the operator site of this operon (16). The finding that none of original mutants especially those with mutations in *marOR *overexpresses *marA *may imply that occurrence of mutation in *marR *is not enough by itself to eliminate MarR repression and complete derepression of *marRAB *operon still needs long exposure to Tc.

On the other hand, it was found that clones C14 and C17 with lower MIC for Tc than clone C6 (Table 1) had *marA *overexpression. This may imply that besides the exposure to Tc, the genetic background is also important for induction of *marRAB *operon. However, it was found that C14 and C17 could not overexpress *acrA *and *micF *(submitted for publication). 

The finding that clone C17 with mutation in operator site of *marRAB *operon could overexpress *marA *is consistent with previous finding obtained for this type of mutation (15). Also it was shown that the replacement of Met with another hydrophobic amino acid (Ile) could cause *marA *overexpression (23). However, the finding that C6 could not overexpress *marA *reveals that substitution of hydrophobic amino acid (Met) with hydrophilic one (Thr) at codon 74 may not provide suitable change for dissociation of MarR from DNA at first and further induction with Tc is needed for complete depression.

In the parallel studies it was found W26 and its clone C14 had a mutation in *acrR *gene encoding AcrR repressor of *acrAB *operon (submitted for publication). This mutation caused alteration of Arg to Cys which was shown to increase the expression of *acrB *(11). However, W26 and C14 did not enhance the expression of *acrA *(submitted for publication). This is confirmed by another finding that W26 and C14 could not tolerate cyclohexane (17 & submitted for publication). It was shown that overexpression of *acrAB-tolC *is necessary for cyclohexane tolerance (24). 

Moreover, overexpression of *acrAB *and *micF *was reported either in mutants with higher MIC for Tc or in the presence of higher concentration of Tc. It was shown that providing these situations caused more *marA *overexpression (21).

Taken together, it is suggested that overexpression of *acrAB *operon and *micF *is not just dependent on overexpression of *marA*. The level of *marA *overexpression is also important for activation of *acrAB *operon and *micF*. This is consistent with the findings of previous work that showed the extent to which genes of the *marA *regulon are activated is a function of MarA concentration (25). Higher levels of *marA *overexpression might gain in clones with the same genetic background and higher MIC for tetracycline. 
